# Macrophage metallothioneins participate in the antileishmanial activity of antimonials

**DOI:** 10.3389/fpara.2023.1242727

**Published:** 2023-10-04

**Authors:** Deninson Alejandro Vargas, David J. Gregory, Roni Nitzan Koren, Dan Zilberstein, Ashton Trey Belew, Najib M. El-Sayed, María Adelaida Gómez

**Affiliations:** 1 Centro Internacional de Entrenamiento e Investigaciones Médicas (CIDEIM), Cali, Colombia; 2 Universidad Icesi, Cali, Colombia; 3 Department of Environmental Health, Harvard T.H. Chan School of Public Health, Boston, MA, United States; 4 Faculty of Biology, Technion-Israel Institute of Technology, Haifa, Israel; 5 Department of Cell Biology and Molecular Genetics, University of Maryland, College Park, MD, United States; 6 Center for Bioinformatics and Computational Biology, University of Maryland, College Park, MD, United States

**Keywords:** metallothioneins, metal transcription factor-1 (MTF-1), *leishmania*, antimony, host-parasite interaction

## Abstract

Host cell functions that participate in the pharmacokinetics and pharmacodynamics (PK/PD) of drugs against intracellular pathogen infections are critical for drug efficacy. In this study, we investigated whether macrophage mechanisms of xenobiotic detoxification contribute to the elimination of intracellular *Leishmania* upon exposure to pentavalent antimonials (Sb^V^). Primary macrophages from patients with cutaneous leishmaniasis (CL) (n=6) were exposed *ex vivo* to *L. V. panamensis* infection and Sb^V^, and transcriptomes were generated. Seven metallothionein (MT) genes, potent scavengers of heavy metals and central elements of the mammalian cell machinery for xenobiotic detoxification, were within the top 20 up-regulated genes. To functionally validate the participation of MTs in drug-mediated killing of intracellular *Leishmania*, tandem knockdown (KD) of MT2-A and MT1-E, MT1-F, and MT1-X was performed using a pan-MT shRNA approach in THP-1 cells. Parasite survival was unaffected in tandem-KD cells, as a consequence of strong transcriptional upregulation of MTs by infection and Sb^V^, overcoming the KD effect. Gene silencing of the metal transcription factor-1 (MTF-1) abrogated expression of MT1 and MT2-A genes, but not ZnT-1. Upon exposure to Sb^V^, intracellular survival of *Leishmania* in MTF-1^KD^ cells was significantly enhanced. Results from this study highlight the participation of macrophage MTs in Sb-dependent parasite killing.

## Introduction

Cutaneous leishmaniasis (CL) is caused by the intracellular protozoan parasite *Leishmania*, and affects more than 1.2 million people annually (Alvar et al., 2012). CL is endemic throughout Central and South America, where control remains dependent on chemotherapy with pentavalent antimonials (Sb^V^). The high rates of treatment failure (as high as 30% in controlled clinical trials), toxicity, and the difficulties associated with access to these drugs limit this control strategy (Romero et al., 2001; Croft et al., 2006; Arevalo et al., 2007; Firdous et al., 2009; Oliveira et al., 2011; Monge-Maillo and López-Vélez, 2013; Sundar and Chakravarty, 2015). The direct activity of antimicrobials against intracellular pathogens is dependent on drug internalization into host cells and on host cell processes that mediate drug metabolism (activation/inactivation). This constitutes a challenge for the design of new drugs and the optimization of existing ones, because host cells act as an additional barrier for drug exposure of the intracellular microbe.

Antimony (Sb) is a metalloid closely related to arsenic (As). Sb exposure induces stress responses leading to activation of mechanisms of redox balance control and metal/xenobiotic detoxification (Cousins, 1994; Roesijadi, 2000; Lima et al., 2010; Coelho et al., 2014). Thus, factors mediating these responses could modulate Sb bioavailability within host cells, impacting the intracellular pharmacokinetics and pharmacodynamics (PK/PD) of these drugs. Illustrating this is evidence of the participation of macrophage ABC transporters in antileishmanial drug effects: Sb modulates the expression of macrophage ATP Binding Cassette (ABC) transporters (Gómez et al., 2014; Barrera et al., 2017). ABCC1, ABCB1 and ABCB5 have been shown to function as Sb efflux pumps in *L. donovani* (Mookerjee Basu et al., 2008) and *L.V. braziliensis* infected macrophages (Téllez et al., 2017), favoring intracellular parasite survival. ABCB6 can function as a plasma membrane Sb efflux transporter and as an intracellular Sb importer, potentially increasing drug concentrations within the phagolysosome, favoring intracellular *L.V. panamensis* killing (Gómez et al., 2014).

Expression of other metal stress responsive genes is also modulated upon Sb exposure (Gómez et al., 2014; Téllez et al., 2017). Among these is metallothionein 2A (MT2-A), a small cysteine-rich cytoplasmic protein involved in zinc homeostasis and the scavenging of metals and electrophilic molecules such as reactive oxygen species (ROS) and nitric oxide (NO) (Nielson et al., 1985; Thornalley and Vašák, 1985; Hamer, 1986; Andrews, 2000; Park et al., 2001; Vašák and Meloni, 2011). There are four MT families (MT1-4): MT1 and MT2 are ubiquitously expressed, whereas MT3 and MT4 are found in the central nervous system and in stratified squamous epithelium, respectively (Hamer, 1986; Namdarghanbari et al., 2011). MTs can bind toxic metals with high affinity such as Cd, Hg, Pd, Ag, As, and Sb (Nielson et al., 1985), resulting in toxic metal tolerance and detoxification (Satoh et al., 1997; Park et al., 2001; Namdarghanbari et al., 2011). This efficient metal scavenging function results from the high thiol content of MTs and the tight regulation of MTs gene expression, which can increase more than 100 fold under metal stress, reaching intracellular protein concentrations of the order of millimolar (Durnam and Palmiter, 1981; Hamer, 1986; Namdarghanbari et al., 2011).

Our group and others have provided evidence for the participation of host MTs in the *Leishmania*-macrophage interactions: a) *Leishmania* infection and Sb^V^ can strongly induce the expression of MT2-A in human macrophages (Gregory et al., 2008; Gómez et al., 2014; Fernandes et al., 2016); b) an inverse correlation of MT2-A gene expression and intracellular survival of *Leishmania* during *in vitro* Sb^V^ exposure has been reported (Gómez et al., 2014); c) an Sb-susceptible *L. V. panamensis* strain induced higher expression of macrophage MT2-A compared with its Sb-resistant counterart, suggesting strain-specific manipulation of MT2-A expression within macrophages (Barrera et al., 2017). However, the role of MTs in their response to antimony and their relationship to antileishmanial drug effects and intracellular parasite survival remains unknown. Based on the above, we sought to dissect and functionally validate the participation of MTs in the Sb-mediated killing of intracellular *Leishmania*.

## Materials and methods

### Ethics statement

This study was approved and monitored by the institutional review board for ethical conduct of research involving human subjects of the Centro Internacional de Entrenamiento e Investigaciones Médicas - CIDEIM, in accordance with national (resolution 008430, República de Colombia, Ministry of Health, 1993) and international (Declaration of Helsinki and amendments, World Medical Association, Fortaleza, Brazil, October 2013) guidelines. All individuals voluntarily participated in the study and written informed consent was obtained from each participant.

### Reagents and chemicals

Additive-free meglumine antimoniate (MA) (Walter Reed 214975AK; lot no. BLO918690-278-1A1W601) was kindly provided by the Walter Reed Army Institute, Silver Spring, MD, USA. Phorbol-12-myristate 13-acetate was purchased from Sigma–Aldrich and zinc acetate dihydrate from J.T. Baker.

### Subjects

Six adult patients, 18 to 65 years of age, with parasitological diagnosis of CL and time of lesion evolution <6 months, without apparent immune deficiencies (negative HIV test, no evidence of immunological disorder nor treatment with medication having immunomodulating effects), participated in this study. For *in vitro* primary macrophage differentiation, peripheral blood mononuclear cells (PBMCs) were obtained from study participants by separation using a Ficoll-Hypaque (Sigma-Aldrich) gradient.

### THP1 and primary macrophage differentiation

The human pro-monocytic cell line THP-1 and derived lines were maintained at 1 x 10^6^ (Firdous et al., 2009) cells/mL in RPMI 1640 supplemented with 10% heat inactivated FBS, 100 μg/mL streptomycin, 100 U/mL penicillin, 5 μg/mL puromycin (only for maintenance of transfected cells lines), at 37°C and 5% CO_2_. THP-1 monocytes were differentiated with 250 ng/mL of PMA for 3 hours, washed twice with D-PBS and cultured 24h in 6 well plates. Human PBMC-derived monocytes were differentiated to macrophages by adherence to cell culture plastic-ware as previously described (Dohmen et al., 2016).

### Parasites, infection and intracellular parasite survival assays

Antimony susceptible *L. (V.) panamensis* promastigotes (MHOM/CO/2002/3594) were kept at 25°C in RPMI supplemented with 10% heat-inactivated FBS, 100 μg/mL streptomycin, 100 U/mL penicillin. Primary human macrophages and differentiated THP-1 cells were infected with human AB+ serum-opsonized stationary phase promastigotes at 10:1 *Leishmania*-macrophage ratio for 2h, washed twice with D-PBS and incubated for 24h at 34°C, 5% CO_2_. After infection was established, cells were exposed for 24h or 48h to MA (8, 16, and 32 μg/mL), or left untreated as a control. Intracellular parasite survival was measured by RT-qPCR as previously described (Romero et al., 2010) (primers used in Supplementary Table S1).

### RNA isolation, cDNA library preparation, and sequence analyses

Total RNA was isolated with Trizol from uninfected, infected, and drug-treated macrophages. RNA quality was assessed with an Agilent 2100 Bioanalyzer using RNAnano chips (Agilent). RNA Integrity Number (RIN) ≥ 7 was considered acceptable to continue with library construction. Poly-A enriched libraries were generated from macrophage RNA extracts using the Illumina TruSeq Standard mRNA preparation kit and checked via the Bioanalyzer and quantitative PCR (KAPA Biosystems). Paired-end reads (100 nt) were obtained using the Illumina HiSeq 1500 (BioProject ID PRJNA633893, Supplementary Table S2). Fastqc [Anon, (2020)] was used to evaluate sequencing quality; Trimmomatic (Bolger et al., 2014) filtered low-quality reads and trimmed bases when the mean quality score fell below a threshold phred score of 20. Reads were mapped against the human (Miga et al., 2014) (hg38) and *L. V. panamensis* (Aslett et al., 2010) genomes (v36ish) using tophat (Trapnell et al., 2012). HTSeq (Anders et al., 2015) was used to count reads mapping to each gene feature. The count tables were restricted to the set of protein coding genes and filtered to remove non-expressed and very weakly expressed genes. The remaining genes were assessed for significant outliers and batch effects by visualizations of normalized data. Library sizes and count densities were calculated on non-normalized data; pairwise correlation and distances, outlier detection, and principal component analysis (PCA) were performed on log_2_, cpm, quantile normalized data with and without accounting for batch in the model or surrogate estimation with sva (using svaseq or combat) (Leek et al., 2012). DESeq2 (Love et al., 2014) was used to perform differential expression analyses alongside a statistically uninformed basic method as a negative control (Supplementary Table S3). Differentially expressed genes were contrasted between control vs. infected and MA-treated primary human macrophages. Genes deemed significantly different according to DESeq2 (∣log_2_FC∣ > 1.0 and a FDR adjusted p-value < 0.05) were passed to various ontology tools. Enrichment PPI network analysis was carried out using STRING 10.

### Short hairpin RNA constructs

A lentivirus-based system was used for shRNA-mediated gene silencing in THP-1 monocytes as previously described (Zhou et al., 2012). At least two independent sets of oligonucleotide pairs for gene knockdown of human MTs and the transcription factor MTF-1 (Supplementary Table S4) were synthesized and cloned into the pLKO.1-TCR vector (Addgene, Cambridge, MA, USA); the same vector was also used as an empty vector control. Lentiviral particles were generated by co-transfection of endotoxin-free hairpin-containing pLKO.1-TCR, psPAX2 and MD2.G (Addgene) into HEK-293T cells. FuGENE HD (Roche) was used as the transfection reagent. Lentivirus-containing cell supernatant was collected 4 days after transfection and subsequently used to transduce THP-1 monocytes in medium containing 10 mg/mL polybrene in a proportion 1:1. Transduced cells were selected under puromycin pressure (5 μg/mL) for a minimum of 5 days. Gene knockdown was confirmed by RT-qPCR using SYBR green (Applied Biosystems) and TaqMan^®^ Gene Expression Assays (Applied Biosystems, Supplementary Table 1). shRNA transduction was confirmed by DNA sequencing.

### Cytotoxicity assays

PMA-differentiated THP-1 cells and derived cell lines were exposed to a dose range (8 μg/mL – 256 μg/mL) of MA for 72 hours. Cell viability was assessed by MTT assay (ATCC) (Supplementary Figure S1).

### Statistical analysis

Based on the distribution of the data, differences in gene expression were tested with one-way analysis of variance and Tukey’s multiple comparisons test. Differences in variance for the remaining experiments were analyzed with unpaired *t* test. A significance level of *p* ≤ 0.05 was used for all statistical tests. Statistical analyses were performed using GraphPad Prism software (version 6).

## Results

### Metallothioneins are within the top 20 macrophages transcripts up-regulated by *Leishmania* infection and exposure to Sb^v^

**FIGURE 1:**
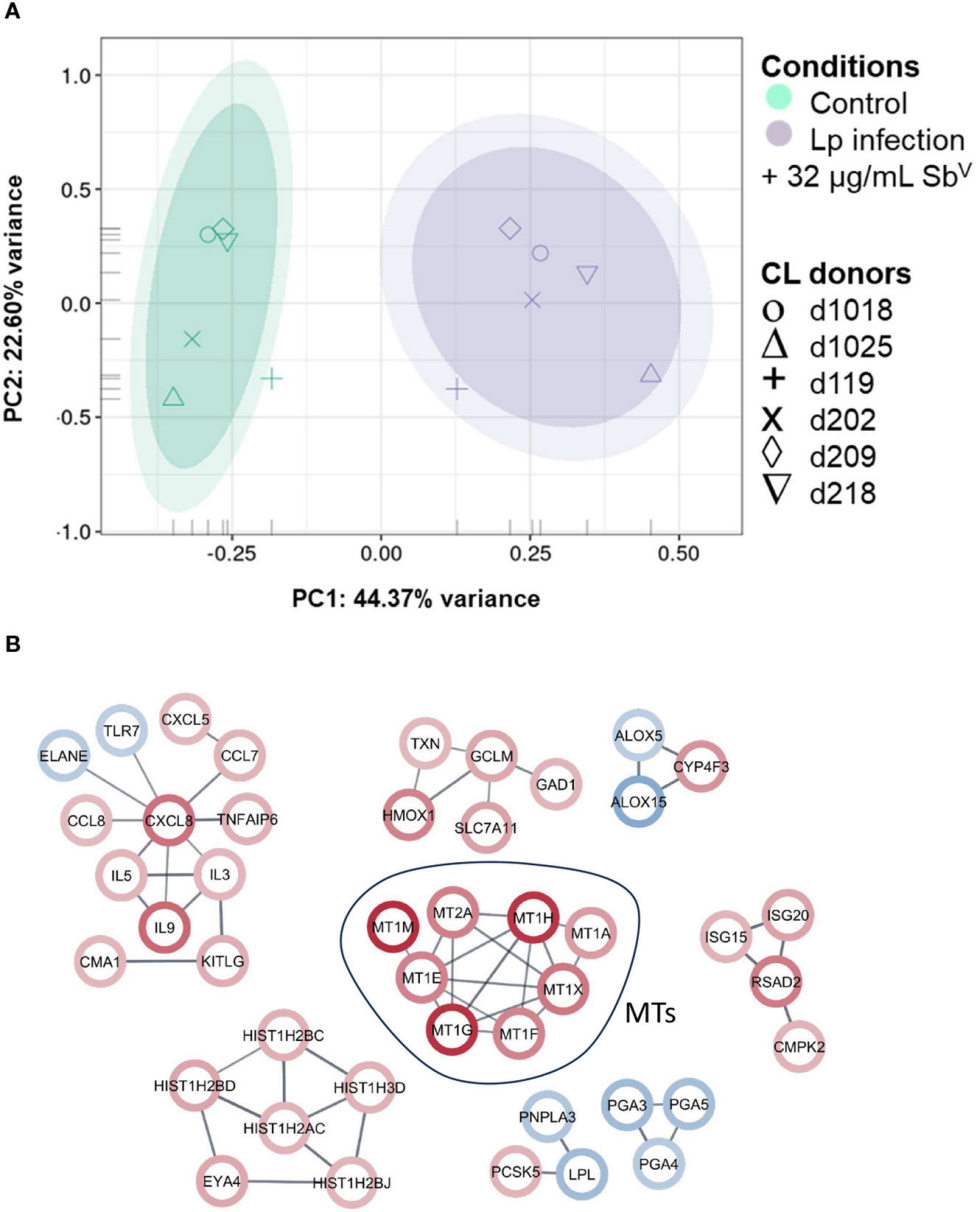
PCA plot and network analysis of macrophage transcriptomes. **(A)** PCA plot of RNA-seq data of primary macrophages from CL patients (n=6), which were infected *in vitro* with *L.V. panamensis* (Lp) and exposed to MA (32μg/mL Sb^V^) for 24 h (purple) or were left uninfected and untreated as controls (green). Ovals represents confidence Interval-CI: 90% and 95% respectively. Each symbol represents each donor. **(B)** STRING network analysis with a ∣logFC∣cutoff ≥ ± 2 and adjusted *p* value ≤ 0.05 (up-regulated genes: red circle; down-regulated genes: blue circle). Confidence of interaction set at 0.7.

Peripheral blood mononuclear cell (PBMC)-derived macrophages from CL patients (n=6) were infected *ex vivo* with *L.V. panamensis* and exposed to Sb^V^ (32μg-Sb/mL, as meglumine antimoniate - MA). Following RNA-seq data collection, a total of 16,841 transcripts were detected. Principal Component Analysis (PCA) showed separation between uninfected/untreated control macrophages and those infected with *L.V. panamensis* and exposed to MA (Figure 1A). After filtering the differential expression (DE) data by ∣logFC∣ ≥ 2 and *p* ≤ 0.05, a set of 217 transcripts remained, of which 111 were up-regulated and 106 down-regulated (Supplementary Table 3). Interestingly, among the top twenty up-regulated transcripts, seven were metallothionein genes: MT1-E, MT1-F, MT1-G, MT1-H, MT1-M, MT1-X and MT2-A (Figure 1B; Supplementary Table 3).

### Induction of MTs expression by zinc acetate enhances MA-mediated killing of *Leishmania*

**FIGURE 2:**
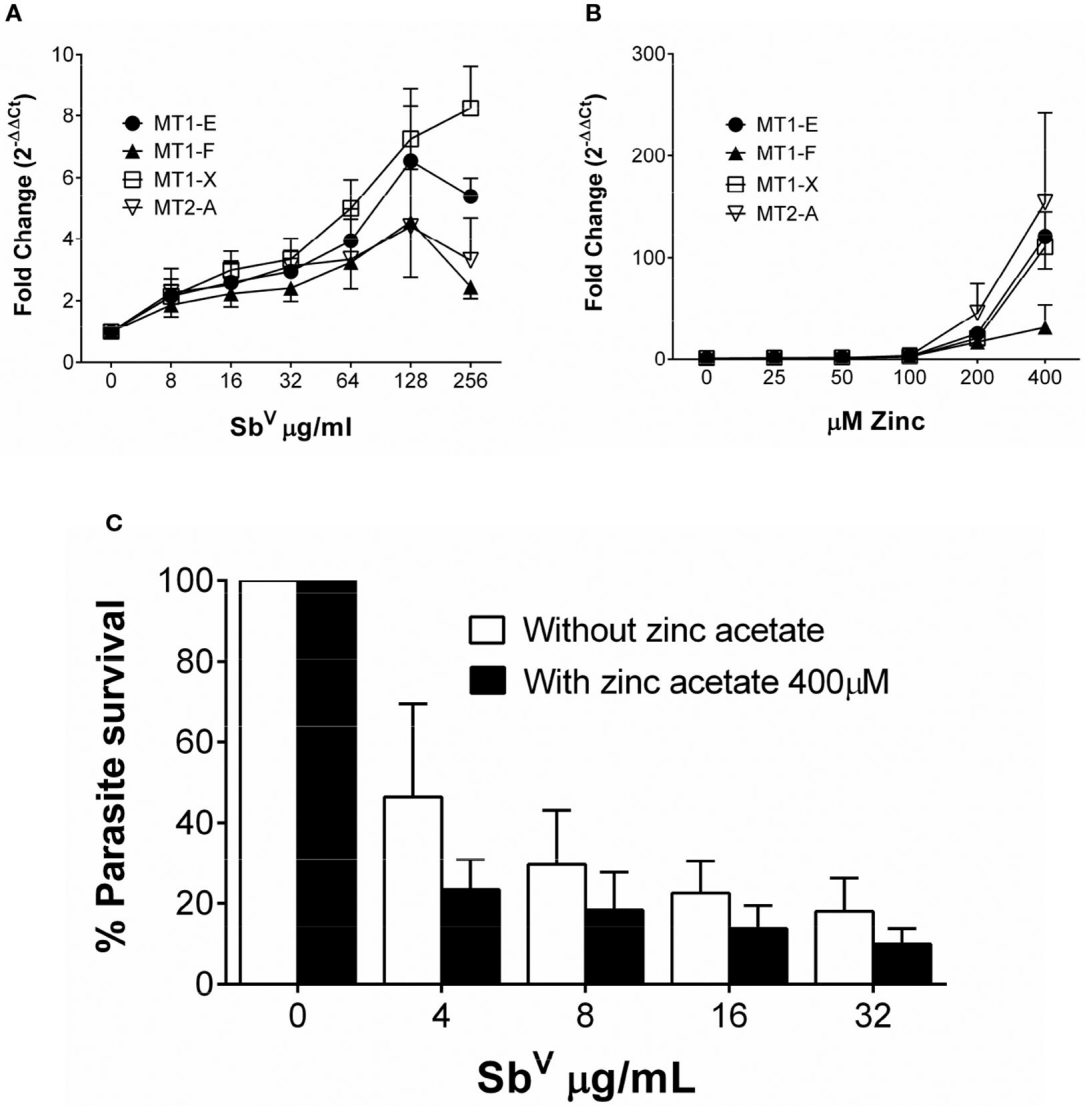
MTs are induced by zinc acetate and antimony. **(A)** Fold change gene expression of MTs in THP-1 cells exposed to sub-cytotoxic doses of Sb^V^. **(B)** Fold change gene expression of MT1-E, 1-F, 1-X and MT2-A in THP-1 cells exposed to increasing doses of zinc acetate. **(C)** *L.V. panamensis* survival (%) in THP-1 cells pre-treated with 400 μM Zn-acetate for 24h and exposed to Sb^V^ (8, 16 and 32 μg/mL). THP-1 gene expression and parasite survival were quantified by RT-qPCR. Each experiment was run as 3 independent replicates. Data are shown as mean ± SD.

To discern how MTs participate in the activity of antimonials, THP-1 cells were exposed to Sb^V^ and the expression of MT1-E, MT1-F, MT1-X, MT2-A, MT3 and MT4 was quantified (primer sequences available in Supplementary Table 1). Expression of MT1-X was the highest, peaking at 8-fold induction over untreated cells; followed by MT1-E, MT2-A and MT1-F (Figure 2A). MT3 and MT4 transcripts were not detected in macrophages.

We evaluated the effect of maximal induction of MTs in intracellular *Leishmania* survival. THP-1 cells were exposed to a dose range of 25 μM to 400 μM Zn acetate for 24h, and peak expression of MTs (>100 fold) was observed with 400 μM Zn acetate (Namdarghanbari et al., 2011) (Figure 2B). Therefore, THP-1 cells were pre-treated for 24h with 400 μM Zn acetate, followed by *L. V. panamensis* infection for additional 24h, and exposed to increasing and non-cytotoxic concentrations of MA (Supplementary Figure 1). Pre-treatment with Zn acetate increased over 40% the Sb-dependent intracellular elimination of *L.V. panamensis* (Figure 2C), supporting the contribution of MTs to this phenotype.

### Strong transcriptional up-regulation of MTs abrogates shRNA silencing of MT genes

**FIGURE 3:**
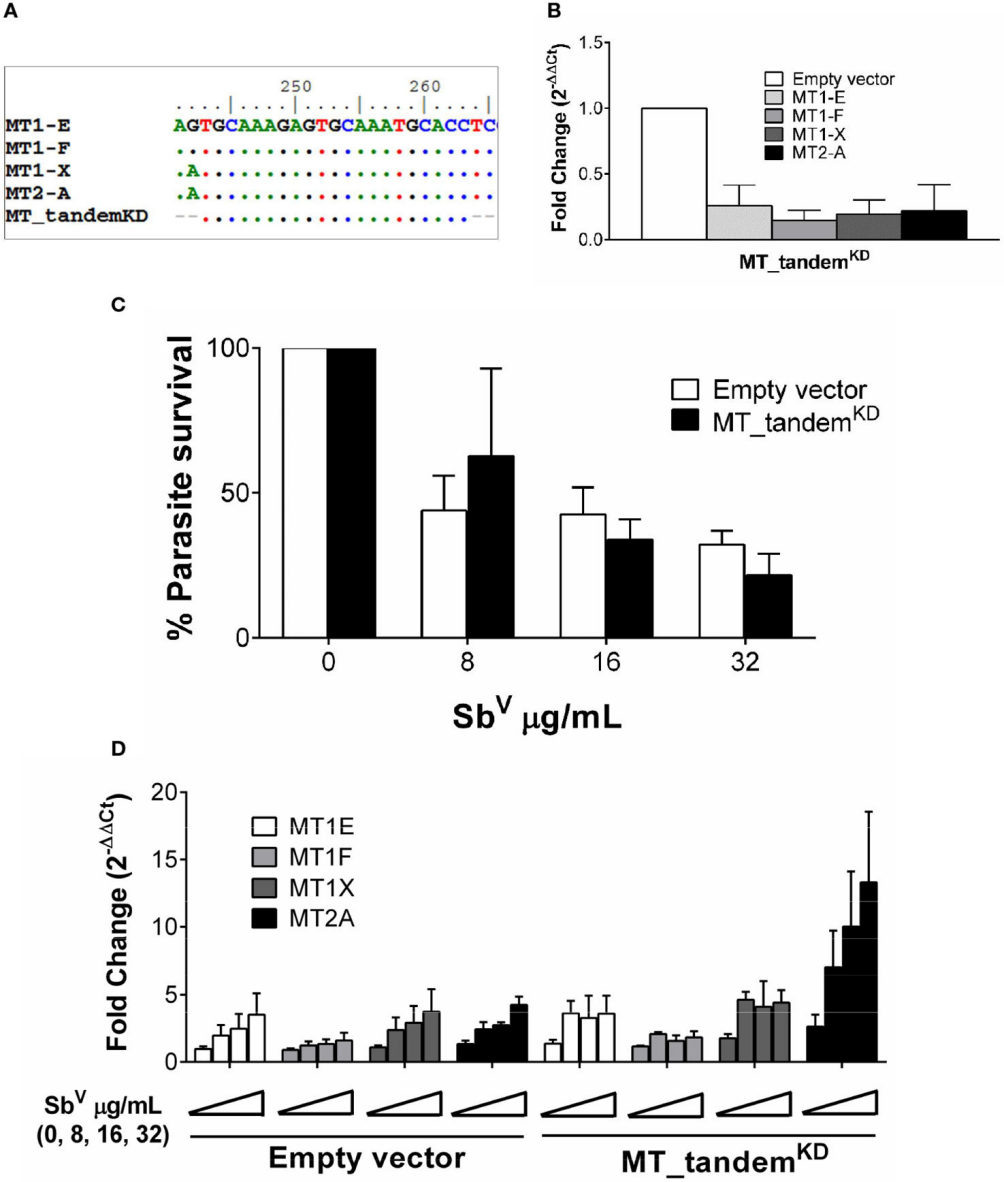
MTs tandem knockdown is abrogated by strong transcriptional up-regulation. **(A)** Sequence alignment of MT genes and MT_tandem^KD^ oligo sequence showing the conserved region targeted for tandem shRNA. **(B)** Validation of the tandem KD shown by MTs gene expression in uninfected MT_tandem^KD^ and empty-vector control cells. **(C)** Percentage of parasite survival in empty vector control and MT_tandem^KD^ cells infected with *L.V. panamensis* and exposed to Sb^V^ (8, 16 and 32 μg/mL). **(D)** Fold change gene expression of MTs upon infection and exposure to Sb^V^ (8, 16 and 32 μg/mL) in empty vector control and MT_tandem^KD^ cells. Gene expression and parasite survival were quantified by RT-qPCR.

An initial assessment of the participation of MT2-A gene silencing on intracellular survival of *Leishmania* did not show any significant effect (Supplementary Figure S2). This led us to hypothesize that a compensatory effect of other MTs maybe operating in our system. Taking advantage of the high sequence similarity of MT genes, an shRNA was constructed which targets the tandem knockdown -KD- (MT_tandem^KD^) of MT1 and MT2 family member genes (Figure 3A). Expression of MT1-E, 1F, 1X and MT2-A was efficiently silenced as shown in Figure 3B. To explore the phenotypic effects of MTs KD on intracellular parasite survival, MT_tandem^KD^ cells were infected with *L. V. panamensis* and exposed to Sb^V^. Despite effective KD of MT genes, intracellular parasite survival remained unchanged compared to empty-vector transfected control cells (Figure 3C). Considering that expression of MT genes is strongly induced by *Leishmania* and Sb^V^, we questioned whether MTs knockdown was maintained during the experimental conditions (infection and drug exposure). Despite the efficient tandem KD of MTs at basal conditions, their strong transcriptional up-regulation during *Leishmania* and Sb^V^ exposure overcame the shRNA-silencing effect (Figure 3D), explaining why parasite survival was similar in MT_tandem^KD^ and empty-vector control cells.

### Expression of MTs is silenced by MTF-1^KD^ and favors survival of intracellular *Leishmania*

**FIGURE 4:**
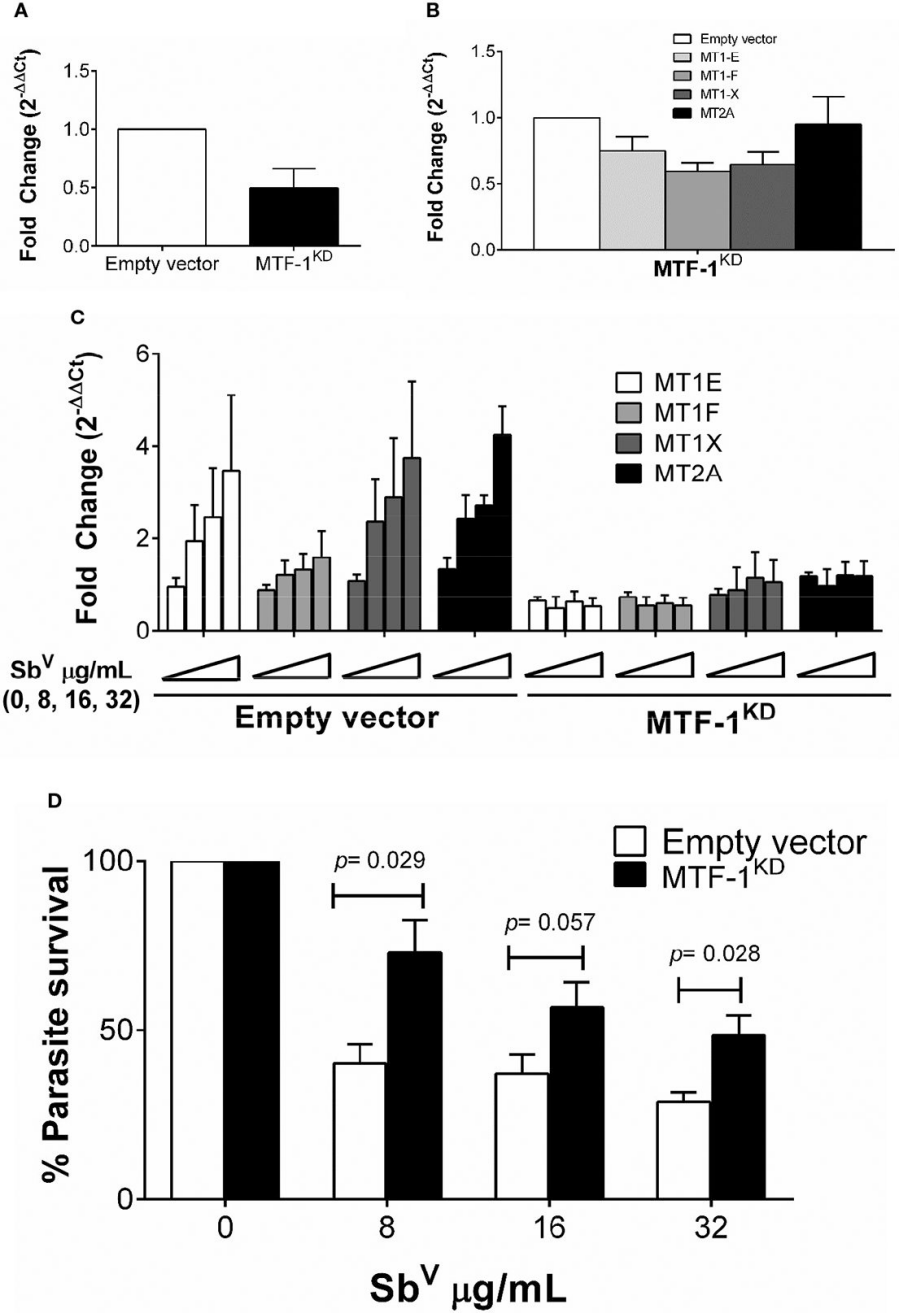
Induction of MTs is abolished by knockdown of MTF-1 favoring intracellular parasite survival. **(A)** Fold change gene expression of MTF-1 in empty vector control and MTF-1KD THP-1 cells. **(B)** Fold change gene expression of MTs in unstimulated or **(C)** infected and SbV (8, 16 and 32 μg/mL) exposed MTF-1KD cells. **(D)** Percentage of intracellular L.V. panamensis survival after SbV exposure. Gene expression and parasite survival were quantified by RT-qPCR. Each experiment was run in 3 independent replicates. Mann-Whitney test was used for statistical analysis. Data are presented as mean values ± SD.

Metal transcription factor-1 (MTF-1) is the principal transcription factor regulating expression of MTs genes during metal-induced stress (Heuchel et al., 1994). We explored whether knockdown of MTF-1 could limit the transcriptional induction of MT genes in our experimental conditions. Using shRNA, the steady-state level of MTF-1 was knocked down by 50% (Figure 4A). Expression of MTs and *slc30a1* (ZnT1) in MTF-1^KD^ cells was evaluated under basal conditions and upon infection with *L.V. panamensis* and exposure to Sb^V^. A slight reduction (ranging from 5% to 40%) of MT1 and MT2 genes expression was observed in uninfected and unstimulated MTF-1^KD^ cells (Figure 4B). However, induction of MT1-E, F, X and MT2-A expression was completely repressed in *L.V. panamensis* infected cells subsequently exposed to Sb (Figure 4C). In the case of *slc30a1* expression, we observed no significant differences in its expression (Supplementary Figure S3), suggesting that the effect of MTF-1^KD^ on MT expression responds from a more specific mechanisms triggered by the presence of xenobiotic metals such as aSb.

MTF-1^KD^ and empty vector control THP-1 cells were infected and exposed to Sb^V^ (8 - 32 μg/mL), and intracellular parasite survival measured by RT-qPCR. Sb-dependent parasite killing was efficient in empty-vector control cells, where parasite survival was below 50% for all Sb^V^ doses tested (Figure 4D). In contrast, *Leishmania* survival after drug exposure significantly increased in MTF-1^KD^ cells compared to empty vector control cells (Figure 4D), and was above 50% for all evaluated doses and up to 75% in the 8 μg/mL Sb^V^ dose. These data suggest that MTF-1^KD^ favors intracellular survival of *Leishmania* by impairing MTs gene expression.

## Discussion

Metallothioneins were initially reported in the early ‘70s and characterized as cadmium binding proteins with a role in metal detoxification (Klaassen et al., 1999). Subsequent studies demonstrated that these proteins could also bind other metals including Sb (Nielson et al., 1985), leading to protection against metal-induced toxicity. Pentavalent antimonials continue to be first line treatment for leishmaniasis in many endemic countries (WHO, 2010). Despite more than 100 years of use, the mechanisms of action and of exposure of the intracellular parasite to the active drug (Sb^III^), remain poorly understood. Recently, the role of host cells in antimony metabolism and detoxification has gained increased attention due to their potential participation in treatment outcome, and as potential targets for optimization of drug exposure (Gómez et al., 2014; Barrera et al., 2017; Téllez et al., 2017). Here, we provide evidence for the participation of macrophage metallothioneins in the Sb-mediated killing of intracellular *Leishmania*.

Studies in MT1/MT2-null mice have demonstrated increased toxicity of cisplatinum, Cd, Hg, Cu, Zn and As -a metalloid closely related to Sb- (Masters et al., 1994; Satoh et al., 1997; Liu et al., 2000; Park et al., 2001; Eddins et al., 2008). Concurring with these findings, tolerance to metal-induced hepato- and nephrotoxicity has been demonstrated in transgenic mice overexpressing MTs (Iszard et al., 1995; Liu et al., 1995; Klaassen et al., 1999). Although the precise mechanism by which MTs facilitate Sb-dependent killing of *Leishmania* remains to be determined, the metal scavenging function of MTs could promote Sb accumulation within infected macrophages. Interestingly, it has been shown that MTs translocate to lysosomes (Moffatt and Denizeau, 1997; Klaassen et al., 1999; Sabolić et al., 2010), suggesting that MT-Sb complexes could be found within lysosomes. *Leishmania* resides in phagolysosomal compartments within host cells. Therefore, as a consequence of the biological process of phagosome-to-phagolysosome maturation, fusion of MT-Sb containing lysosomes with *Leishmania*-containing phagosomes could result in enhanced exposure of the intracellular parasite to the drug.

Pentavalent antimonials are pro-drugs which need to be reduced to the trivalent active form to exert their antileishmanial activity (Sb^V^→Sb^III^). MTs have an important function in the cellular redox balance due to their high thiol content (Nielson et al., 1985; Thornalley and Vašák, 1985; Hamer, 1986; Andrews, 2000; Park et al., 2001; Vašák and Meloni, 2011). Under stress conditions, the strong up-regulation of MTs gene expression results in a redox capacity that can surpass that of GSH (Durnam and Palmiter, 1981; Hamer, 1986; Namdarghanbari et al., 2011). High GSH content has been shown to promote Sb^V^ to Sb^III^ reduction in mammalian cells as well as in *Leishmania* (Miekeley et al., 2002; Dos Santos Ferreira et al., 2003). Thus, MTs could participate in the reduction Sb^V^ to Sb^III^ favoring parasite elimination.

MTF-1 is the main transcription factor involved in MTs expression during metal stress responses, and partially during oxidative stress exposure/response (Dalton et al., 1999; Andrews, 2000; Pearce et al., 2000; St. Croix et al., 2002; Stitt et al., 2006). Our results and those of others demonstrate that MTF-1 knockout/knockdown efficiently abolishes cellular expression of MTs under metal and oxidative stress (Heuchel et al., 1994; Andrews, 2000; St. Croix et al., 2002). Although our data provide evidence that repression of MTs expression via MTF-1 gene knockdown promotes intracellular survival of *Leishmania* after exposure to Sb, we cannot rule out the intervention of other MTF-1 mediated mechanisms in the enhanced parasite survival, such as those involved in Zn transport (Lichtlen et al., 2001; Laity and Andrews, 2007; Troadec et al., 2010; Lichten et al., 2011; Kim et al., 2014). However, evaluation the effect of MTF-1 KD in the expression of *slc30a1* (gene coding for the zinc transporter ZnT1) known to be modulated by MTF-1, did not result in significant expression differences in control and MTF-1^KD^ cells when infected and exposed to Sb. We hypothesize that the specific effect observed over MT genes responds to a xenobiotic metal-specific response, which is supported by four MTF-1 binding sites in MT promoter regions, making this transcription factor the primary transcriptional regulator of MT genes. As was the case of ZnT1, and likely other genes where MTF-1 has some transcriptional participation, other transcription factors could be compensating for their expression in absence of MTF-1. However this remains to be experimentally demonstrated.

Our results support a dual role of MTs in toxic as well as therapeutic metal binding, highlighting the potential to harness host cell redox and metal detoxification systems to enhance drug bioavailability and exposure targeted to intracellular pathogens. These findings enlighten interesting drug-related homeostatic processes occurring during treatment of intracellular microbe infections, whereby the same mechanism that promotes host protection to drug-induced toxicity, in this case against Sb-induced stress, can enhance the antimicrobial activity of the drug.

## Supplementary Material

Datasheet_1

Table

## Data Availability

The original contributions presented in the study are publicly available. This data can be found here: https://www.ncbi.nlm.nih.gov/bioproject/?term=PRJNA633893.
