# Supplementary material for: Macrophage metallothioneins participate in the antileishmanial activity of antimonials
Source: Front Parasitol. Author manuscript; Available in PMC 2024 Jan 18. (PMC10795579; doi:10.3389/fpara.2023.1242727)
Supplement: Datasheet_1 [file NIHMS1955141-supplement-Datasheet_1.docx]

**Supplementary table 1. Primers used for gene knockdown confirmation and quantification of mammalian and *Leishmania* cells.**

| **Primer name** | **Primer sequence 5'-->3'** | **Gen** | **Fluorofore** |
| --- | --- | --- | --- |
| GAPDH | Hs99999905_m1 | GAPDH | FAM  TaqMan® |
| TATA_F | CACGAACCACGGCACTGATT | TBP | SYBR Green |
| TATA_R | TTTTCTTGCTGCCAGTCTGGAC |  | SYBR Green |
| 7SLRNA_F | TGCTCTGTAACCTTCGGGGGCT | Leishmania 7SLRNA | SYBR Green |
| 7SLRNA_R | GGCTGCTCCGTYNCCGGCCTGACCC |  | SYBR Green |
| MT4 | [Hs00262914_m1](https://www.thermofisher.com/taqman-gene-expression/product/Hs00262914_m1?CID=&ICID=&subtype=) | MT4 | FAM  TaqMan® |
| MT3 | [Hs01921768_s1](https://www.thermofisher.com/taqman-gene-expression/product/Hs01921768_s1?CID=&ICID=&subtype=) | MT3 | FAM  TaqMan® |
| MT2-A | Hs01591333_g1 | MT2-A | FAM  TaqMan® |
| MT1-E_F | GCATCCCCTTTGCTCGAAAT | MT1-E | SYBR Green |
| MT1-E_R | CAGCTGCACTTCTCCGATG |  | SYBR Green |
| MT1-F_F | CCCCCTCCCCTGACTATCAA | MT1-F | SYBR Green |
| MT1-F_R | AGGAGCAGCAGCTCTTCTTG |  | SYBR Green |
| MT1-X_F | CTGCTTCTCCTTGCCTCGAA | MT1-X | SYBR Green |
| MT1-X_R | TGTCTGACGTCCCTTTGCAG |  | SYBR Green |
| MTF-1_F | GCCATTTGAGTGTGACGTGC | MTF-1 | SYBR Green |
| MTF-1_R | CATCGTGATCGCACCGAAAT |  | SYBR Green |

**Supplementary table 2. Accession numbers of sequences uploaded. BioProject ID** [**PRJNA633893**](https://www.ncbi.nlm.nih.gov/bioproject/?term=PRJNA633893)

| **Accession** | **Title/Sample name** | **Donor** | **Condition (24h)*** |
| --- | --- | --- | --- |
| SRR12185387 | HPGL0315 | d202 | None |
| SRR12185386 | HPGL0316 | d202 | Lp+Sb^V^ |
| SRR12185383 | HPGL0317 | d209 | None |
| SRR12185382 | HPGL0318 | d209 | Lp+Sb^V^ |
| SRR12185381 | HPGL0319 | d218 | None |
| SRR12185380 | HPGL0320 | d218 | Lp+Sb^V^ |
| SRR12185379 | HPGL0321 | d1019 | None |
| SRR12185378 | HPGL0322 | d1019 | Lp+Sb^V^ |
| SRR12185377 | HPGL0640 | d1025 | None |
| SRR12185376 | HPGL0641 | d1025 | Lp+Sb^V^ |
| SRR12185385 | HPGL0642 | d1018 | None |
| SRR12185384 | HPGL0643 | d1018 | Lp+Sb^V^ |

***Conditions:**

**None:** uninfected macrophages unexposed to drugs.

**Lp+Sb^V^**: Macrophages infected for 24h with *Leishamania panamensis* (MOI 1:10), followed by 24h of exposure to 32 µg/mL Sb^V^.

**Supplementary table 4. Oligonucleotide pairs for gene knockdown.**

In bold an underline, restriction site for Xho1.

| **Oligo name** | **Oligos sequence 5'-->3' (shRNA)** | **Gen knocking down** |
| --- | --- | --- |
| MT2-A_Top1 | CCGGCTGCAAATGCAAAGAGTGCAA**CTCGAG**TTGCACTCTTTGCATTTGCAGTTTTTTG | MT2-A |
| MT2-A_Bottom1 | AATTCAAAAAACTGCAAATGCAAATCGTGCAA**CTCGAG**TTGCACTCTTTGCATTTGCAG |  |
| MT2-A_Top2 | CCGGGCAAAGAGTGCAAATGCACTT**CTCGAG**AAGTGCATTTGCACTCTTTGCTTTTTTG |  |
| MT2-A_Bottom2 | AATTCAAAAAAGCAAAGAGTGCAAATGCACTT**CTCGAG**AAGTGCATTTGCACTCTTTGC |  |
| MT_Tandem_Top1 | CCGGTGCAAAGAGTGCAAATGCACC**CTCGAG**GGTGCATTTGCACTCTTTGCATTTTTG | MTs-tandem |
| MT _Tandem_Bottom1 | AATTCAAAAATGCAAAGAGTGCAAATGCACC**CTCGAG**GGTGCATTTGCACTCTTTGCA |  |
| MT_Tandem_Top2 | CCGGAATGCACCTCCTGCAAGAAA**CTCGAG**TTTCTTGCAGGAGGTGCATTTTTTTG |  |
| MT_Tandem_Bottom2 | AATTCAAAAAAATGCACCTCCTGCAAGAAA**CTCGAG**TTTCTTGCAGGAGGTGCATT |  |
| MTF-1_Top1 | CCGGCAGAACTTACAATGGATATTA**CTCGAG**TAATATCCATTGTAAGTTCTGTTTTTTG | MTF-1 |
| MTF-1_Bottom1 | AATTCAAAAAACAGAACTTACAATGGATATTA**CTCGAG**TAATATCCATTGTAAGTTCTG |  |
| MTF-1_Top2 | CCGGCTCACCAGATCAGATTCATTT**CTCGAG**AAATGAATCTGATCTGGTGAGTTTTTTG |  |
| MTF-1_Bottom2 | AATTCAAAAAACTCACCAGATCAGATTCATTT**CTCGAG**AAATGAATCTGATCTGGTGAG |  |


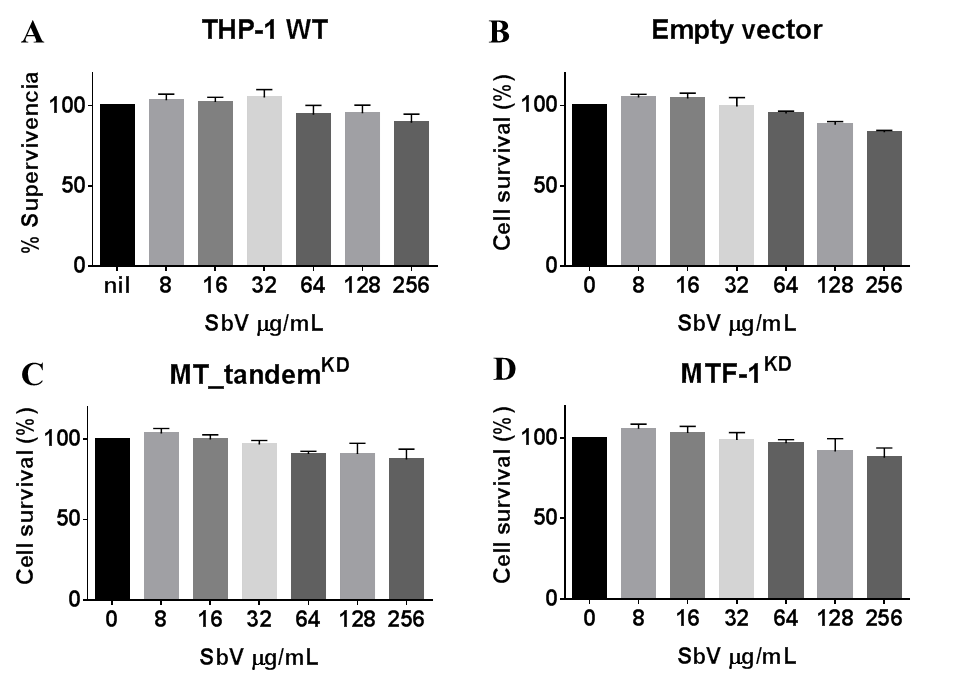


***Supplementary figure 1. Cytotoxicity assay of THP-1 cell lines.*** Wild type cells (**A**), empty vector-transfected controls (**B**), MT_tandem^KD^ (**C**), and MTF-1^KD^ cells (**D**), were exposed to increasing doses of meglumine antimoniate (Sb^V^ 8 to 256 µg/mL) for 48h at 37 °C 5% CO_2_ and cytotoxicity was evaluated by the MTT method. Each experiment was run in 3 independent replicates. Data are shown as average ± SD.


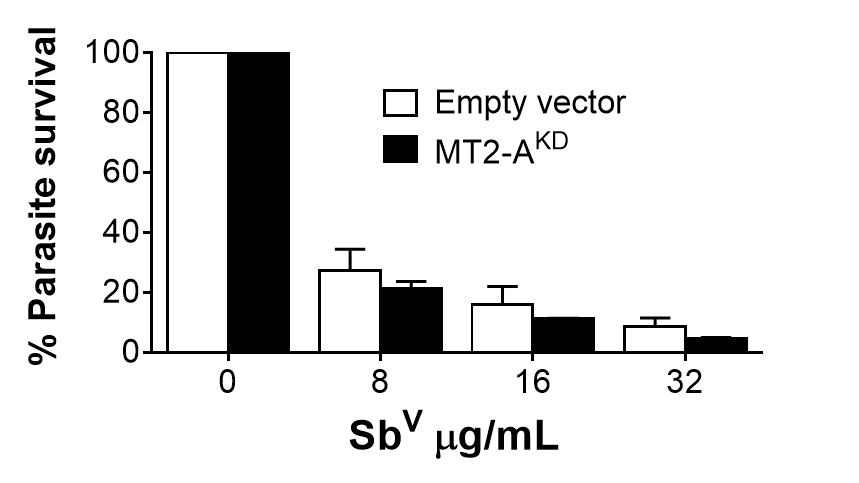


**Supplementary figure 2.** Intracellular *Leishmania* survival in MT2-A knockdown THP-1 derived macrophages. Empty vector (control) and knockdown (MT2-A KD) cells were infected with *L. panamensis* (transfected with luciferase) and exposed to different concentrations of meglumine antimoniate for 48h. Parasite survival was measured by luciferase activity in relative light units. Data are shown as percent survival relative to the drug-free control. Data represent the mean value ± SEM of three biological replicas.

***Supplementary figure 3.*** *Gene expression of the zinc transporter 1 -ZnT1-.* Empty vector control and knockdown (MTF-1 KD) cells were infected with *L. panamensis* and exposed to different concentrations of meglumine antimoniate for 48h. ZnT1 expression was evaluated by RT-PCR using Taqman probes. Data represent the mean value ± SD of three biological replicas.
